# An adjuvanted respiratory syncytial virus fusion protein induces protection in aged BALB/c mice

**DOI:** 10.1186/1742-4933-9-21

**Published:** 2012-10-02

**Authors:** Anu Cherukuri, Kate L Stokes, Kathryn Patton, Howard Kuo, Kaori Sakamoto, Stacie Lambert, Elizabeth Stillman, Martin L Moore, Sujin Lee

**Affiliations:** 1 Infectious Diseases/Vaccines Research, MedImmune, LLC, Mountain View, CA, USA; 2 Department of Pediatrics, Emory Children’s Center, Emory University, Atlanta, GA, USA; 3 Children’s Healthcare of Atlanta, Atlanta, GA, USA; 4 Department of Pathology, College of Veterinary Medicine, University of Georgia, Athens, GA, USA

**Keywords:** Respiratory Syncytial Virus, Immunosenescence, Alum, Adjuvant, Aged mice

## Abstract

**Background:**

Respiratory Syncytial Virus (RSV) causes significant disease in the elderly, in part, because immunosenescence impairs protective immune responses to infection in this population. Despite previous and current efforts, there is no RSV vaccine currently licensed in infants or elderly adults. Adjuvanted RSV subunit vaccines have the potential to boost waning immune responses and reduce the burden of RSV disease in the elderly population.

**Results:**

We used an aged BALB/c mouse model to evaluate immune responses to RSV Fusion (F) protein in the absence and presence of an alum adjuvant. We demonstrate that aged BALB/c mice immunized with alum-adjuvanted RSV F protein had significantly reduced lung viral titers at day 4 following challenge with wild-type (*wt*) RSV. Serum neutralizing antibody titers measured on day 27 correlated with protection in both young and aged vaccinated mice, although the magnitude of antibody titers was lower in aged mice. Unlike young mice, in aged mice, alum-adjuvanted RSV F did not induce lung T_H_2-type cytokines or eosinophil infiltration compared to non-adjuvanted F protein following *wt* RSV challenge.

**Conclusion:**

Our studies demonstrate that neutralizing anti-RSV antibody titers correlate with protection in both young and aged BALB/c mice vaccinated with RSV F protein vaccines. The F + alum formulation mediated greater protection compared to the non-adjuvanted F protein in both young and aged mice. However, while alum can boost F-specific antibody responses in aged mice, it does not completely overcome the reduced ability of a senescent immune system to respond to the RSV F antigen. Thus, our data suggest that a stronger adjuvant may be required for the prevention of RSV disease in immunosenescent populations, to achieve the appropriate balance of protective neutralizing antibodies and effective T_H_1-type cytokine response along with minimal lung immunopathology.

## Background

Respiratory Syncytial Virus (RSV) is the most important viral pathogen responsible for lower respiratory tract illness in infants, and a major cause of morbidity and mortality in the elderly [1,2]. In elderly patients, RSV caused 11% of hospitalizations for pneumonia [1,3]. The overwhelming majority of RSV-associated deaths in the USA are in the elderly [1,4,5]. To date, there is no approved vaccine to prevent RSV disease and the requisite components for vaccine efficacy against RSV disease in the elderly are largely unknown. Immunosenescence is a hallmark of aging and impairs the capacity to respond to vaccination, as well as the ability to prevent infection. Mechanisms of immunosenescence that result in weaker responses to vaccination in the elderly are not well understood. We undertook this study because evaluation of vaccine responses in aged mouse models may be informative in understanding vaccine-induced immune responses in the elderly.

Purified RSV fusion (F), attachment (G), and matrix (M) proteins have been developed as subunit vaccines [6,7]. RSV fusion (F) protein has been tested in the clinic as a potential subunit vaccine [6,7], and one vaccine (PFP-2) was tested in adults over age 60 [8,9]. However, RSV subunit vaccines have been plagued with poor immunogenicity in the elderly [10,11]. In addition, it has been shown that immunization with purified F protein enhanced pulmonary histopathology in cotton rats and mice [12,13]. Thus, it appears that RSV F protein alone is insufficient to show protection in aged mice due to poor immunogenicity. We hypothesized that adjuvant mixed with F protein may overcome poor immunogenicity, reduce histopathology, and induce protection in aged mice. We chose the aluminum salts (alum) as an adjuvant to test our hypothesis. Until recently, alum was the only adjuvant licensed for human use in the USA [14]. Alum-adjuvanted RSV vaccines have been evaluated in elderly humans and in mouse models but the adjuvant effects of alum were not consistent. A study published by Falsey *et al.* showed no adjuvanting effect of alum on RSV F or RSV G protein vaccines in the elderly [15]. Langley *et al.* reported that an alum-containing RSV subunit vaccine had decreased immunogenicity compared to non-adjuvanted F in the elderly [16]. These observations could be the result of: 1) differences in the quality of the F antigen used; 2) poor suitability of alum as an adjuvant for a booster vaccine in an RSV-seropositive population; or 3) poor performance of the alum-adjuvanted RSV F vaccine in a setting of immunosenescence. Our current study focuses on the third possibility. We sought to evaluate the immunogenicity of a pure, recombinant RSV F protein adjuvanted with alum in the context of immunosenescence by utilizing an aged BALB/c mouse model. The BALB/c mouse model was chosen since this inbred strain is relatively susceptible to RSV infection [17].

We demonstrate that neutralizing anti-RSV antibody titers correlated with protection in young and aged BALB/c mice vaccinated with RSV F protein vaccines. The F + alum formulation protected to a greater degree than non-adjuvanted F in both young and aged mice. Notably, F antigen alone induced T_H_2-type responses and eosinophil infiltration in both young and aged mice, and the addition of alum alleviated lung T_H_2-type cytokine responses and eosinophil infiltration compared to the non-adjuvanted F group. However, aged mice achieved lower levels of neutralizing antibodies and less protection than young mice given the same dose of vaccine. While T_H_2 responses were lower in aged mice compared to young mice, the decreased neutralizing antibodies and protection suggest that immune responses were generally diminished in the aged mice compared to the young mice. Importantly, alum did not induce inflammatory chemokines or enhanced immunopathologic lesions following *wt* RSV challenge in the lung airways of aged mice as was observed in young mice.

## Results

### Effect of alum on RSV F protein vaccine efficacy in young and aged mice

**Figure 1:**
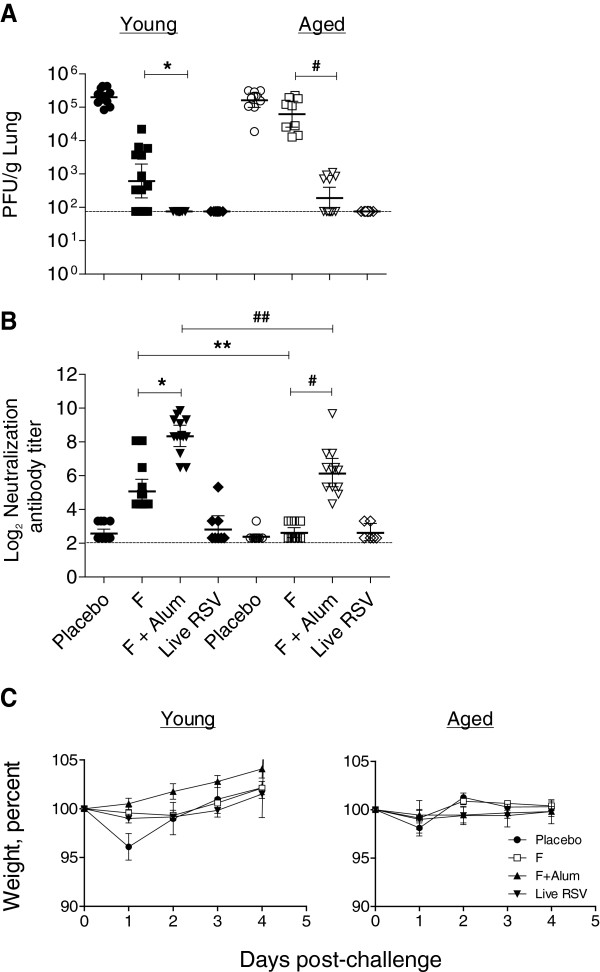
**Lung viral loads and serum neutralizing antibodies in vaccinated young and aged BALB/c mice.** Groups of mice (n=14) were vaccinated twice i.m. and sera were collected by submandibular bleeding at day 27. Mice were challenged intranasally with 10^6^ PFU RSV strain A2 at day 28. (**A**) Viral load. Lungs were harvested 4 days post-challenge, homogenized, and infectious RSV was titrated by plaque assay. *P < 0.05 comparing F to F + alum in young mice (ANOVA). ^#^, *P* < 0.05 comparing F to F + alum in aged mice (ANOVA). Each symbol represents an individual mouse. The dotted line represents the limit of detection. (**B**) Neutralizing antibody titer. Sera from vaccinated mice were tested for RSV neutralizing activity, *P < 0.05 comparing F to F + alum in young mice (ANOVA). ^#^, *P* < 0.05 comparing F to F + alum in aged mice (ANOVA). **, *P* < 0.05 comparing F in young mice to F in aged mice (ANOVA). ^##^, *P* < 0.05 comparing F + alum in young mice to F + alum in aged mice (ANOVA). The dotted line represents the limit of detection. (**C**) Body weight. Groups of mice (n=14) were unvaccinated or vaccinated twice i.m. and challenged intranasally with 10^6^ PFU RSV strain A2 at day 28. Body weights were recorded daily for 4 days. Data are combined from two independent experiments.

In both young and aged mice, live RSV-infected mice were fully protected from challenge (Figure 1A). A dose titration of RSV F protein ranging from 0.03 – 3 μg was previously performed in young mice in our laboratory and demonstrated that partial protection against *wt* RSV challenge was achieved with a 0.3 μg dose of F (data not shown). Therefore, we chose 0.3 μg of RSV F as an immunization dose in this study to evaluate and compare the effects of adjuvanting F with alum on mediating protection in young and aged mice. Young mice vaccinated with non-adjuvanted F alone showed partial viral clearance, while no detectable lung viral load was found in the F + alum vaccinated group or the live RSV group (Figure 1A). In contrast, aged mice receiving non-adjuvanted F alone were not protected, and aged mice receiving F + alum showed partial protection (Figure 1A). Notably, F + alum vaccine significantly reduced viral load compared to non-adjuvanted F vaccine in both young and aged mice.

To determine the immune correlates of protection against challenge following vaccination, we measured the day 27 serum neutralizing antibody titers in young and aged mice (Figure 1B). The neutralizing antibody titers demonstrated a strong inverse correlation with lung viral titers following RSV infection (r^2^=−0.837). In the young mice, complete protection was observed in the F + alum group, which also showed the highest mean neutralizing antibody titer of 8.0 log_2_ (Figure 1A and 1B). A neutralizing antibody titer ≥ 5.82 log_2_ in the F or F + alum-vaccinated groups appeared to correlate with lung protection (Figure 1A and 1B). While alum enhanced the neutralizing antibody titers in both the young and aged mice, the neutralizing antibody titers in young mice were of a higher magnitude than that observed in aged mice (Figure 1B). There were significant differences in neutralizing antibody titers between the young and aged mice vaccinated with non-adjuvanted F alone (** = *P* < 0.005, ANOVA) and between the young and aged F + alum-vaccinated mice (## = *P* < 0.005, ANOVA) (Figure 1B). In contrast, low neutralizing antibody titers were detected in the live RSV + RSV challenge mice. This might be due to timing of sampling [18] or alternatively, point to a role for T cells and/or mucosal antibodies in mediating protection during live RSV infection. We measured body weight as an indicator of RSV illness severity following RSV challenge [19]. Weight loss was detected only in the placebo group in young mice at day 1 following RSV challenge (*P* = 0.006) (Figure 1C). This result indicated that non-adjuvanted F and F + alum vaccination followed by *wt* RSV challenge did not result in exacerbation of illness as measured by weight loss. Taken together, these data support the conclusion that while alum can boost F-specific antibody responses in aged mice, it does not completely overcome the reduced ability of a senescent immune system to respond to an antigen.

### Alum-adjuvanted F protein induces higher RSV F-specific antibody response compared to non-adjuvanted F protein in young and aged mice

**Figure 2:**
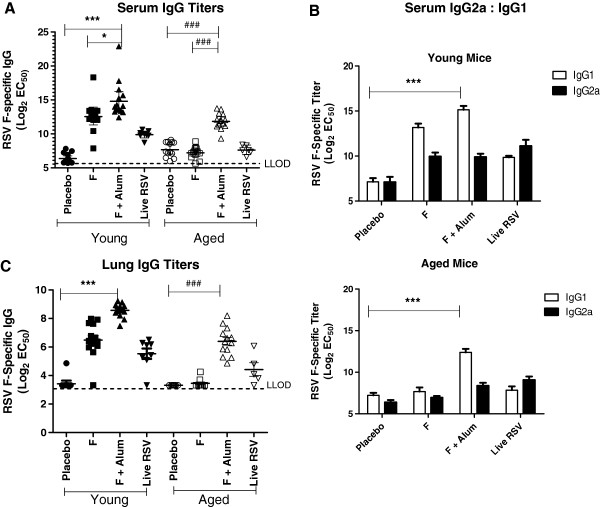
**RSV F-specific serum and lung antibody titers in vaccinated young and aged BALB/c mice.** Day 27 serum was examined by ELISA for (**A**) RSV F-binding IgG and (**B**) RSV F-binding IgG2a or IgG1 subtype antibodies using isotype-specific anti-mouse detection antibodies. (**C**) Lung homogenates 4 days post-challenge were examined for RSV F-binding IgG antibodies. The dotted line represents the lower limit of detection (LLOD= 1:50 dilution). ***, *P* < 0.0001 comparing F + alum to placebo in young mice (ANOVA). *, *P* < 0.05 comparing F + alum to F in young mice (ANOVA). ###, *P* < 0.0001 comparing F + alum to placebo or F in aged mice (ANOVA). Serology results were combined from two independent experiments.

Next, we measured RSV F-specific antibody responses in serum and lung homogenates. Overall, the mean serum and lung IgG titers observed in the young mice were higher than that observed in aged mice (Figure 2). Both young and aged mice immunized with F + alum had significantly higher serum RSV F-specific IgG antibody titers compared to placebo or F vaccine groups (*P* < 0.0001, ANOVA) (Figure 2A). Non-adjuvanted F and F + alum vaccines induced higher levels of RSV F-specific IgG1 compared to IgG2a in young mice (Figure 2B). The data are consistent with previous observations that alum drives a primarily T_H_2-biased antibody response [16,20]. Regardless of the IgG isotype, we observed that the serum RSV F-specific IgG titers correlated with protection in mice vaccinated with RSV F protein. Live RSV infection induced equivalent titers of RSV F-specific IgG1 and IgG2a in young mice, while in aged mice the IgG subtype titers were near the limit of detection. In addition, we measured both RSV F-specific IgG and IgA antibodies in the lung homogenates; however, only the IgG antibodies were present at detectable levels in all groups and reported here (Figure 2C). RSV F-specific IgA antibodies were detected only in the live RSV-infected group of both young (5.0 ± 0.8 log_2_ EC_50_ titer) and aged mice (4.0 ± 1.2 log_2_ EC_50_ titer) (data not shown). The RSV F-specific lung IgG antibodies induced by the different vaccine formulations (Figure 2C) displayed an inverse correlation with the lung viral titers shown in Figure 1A. Vaccinating with F + alum resulted in lung IgG antibody titers with the highest magnitude compared to the other vaccine groups regardless of the age of the mice (Figure 2C). 

### Distinct T_H_1- and T_H_2-type cytokine profiles induced in the lungs of young and aged mice

**Figure 3:**
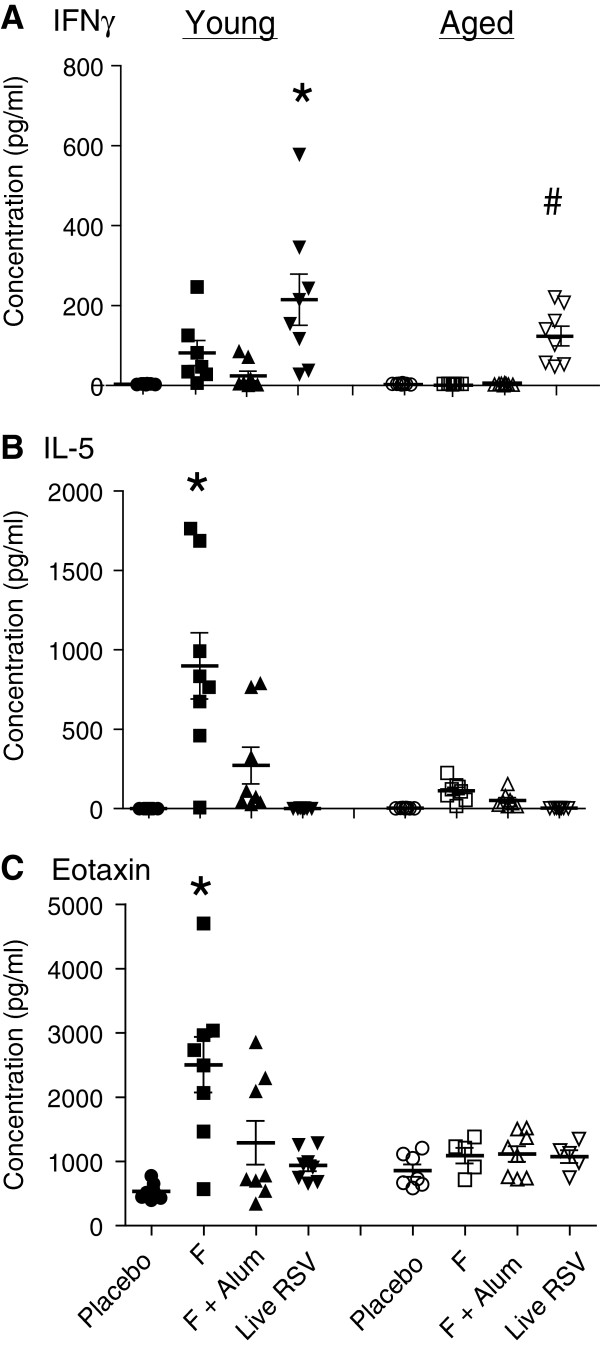
**Cytokine levels in the lung homogenates.** Cytokines and chemokines in lung homogenates isolated 4 days post-challenge were measured using a 15-plex bead array panel (Luminex). Shown are representative T_H_1 (IFNγ), T_H_2 (IL-5) cytokines, and eotaxin levels. (**A**) IFNγ levels. *, *P* < 0.05 comparing RSV to placebo, F, or F + alum in young mice (ANOVA). ^#^, *P* < 0.05 comparing RSV to placebo, F, or F + alum in aged mice (ANOVA). (**B**) IL-5 levels. *, *P* < 0.05 comparing F to F + alum in young mice (ANOVA). (**C**) Eotaxin levels. *, *P* < 0.05 comparing RSV to placebo, F, or F + alum in young mice (ANOVA).

To investigate the T_H_1- and T_H_2-type immune responses after vaccination and RSV challenge, production of IFN-γ (T_H_1 cytokine) and IL-5 (T_H_2 cytokine) were measured in lung homogenates (Figure 3A and Figure 3B). Overall, significantly lower levels of IFN-γ and IL-5 were induced in vaccinated aged mice compared with vaccinated young mice. Interestingly, only live RSV induced IFN-γ in both young and aged mice (P < 0.05) (Figure 3A). High levels of IL-5 were detected in young mice vaccinated with non-adjuvanted F or F + alum (Figure 3B). In contrast, we detected basal levels of IL-5 in aged mice (Figure 3B). As IL-5 is implicated in contributing to eosinophilia, we also measured lung eotaxin (CCL11), a chemoattractant that promotes eosinophil recruitment to the lungs [21,22]. The levels of eotaxin in young mice immunized with non-adjuvanted F were significantly higher than the levels in the other groups (Figure 3C). Relatively low levels of eotaxin were observed in the lungs of all aged mice treatment groups (Figure 3C). The T_H_2 cytokine IL-13, which was also included in our cytokine multiplex panel was detected at low levels (250 ± 60 pg/mL) only in young mice immunized with non-adjuvanted F. IL-13 levels were below the detection limit in aged mice of all treatment groups (data not shown). Levels of the T_H_2 cytokine, IL-4, were below the limit of detection of the cytokine multiplex assay (data not shown). Among the chemokines included in our experiments, the only two that were induced to a measurable extent were KC (CXCL1) and MCP-1 (CCL2) in the non-adjuvanted F vaccinated young mice (2650 ± 1250 pg/mL and 800 ± 300 pg/mL, respectively). In contrast, non-adjuvanted F vaccinated aged mice did not show a measurable induction of KC or MCP-1 in the lungs following viral challenge (data not shown). 

### Differential eosinophil abundance induced in the lungs of young and aged mice following vaccination and *wt* RSV challenge

**Figure 4:**
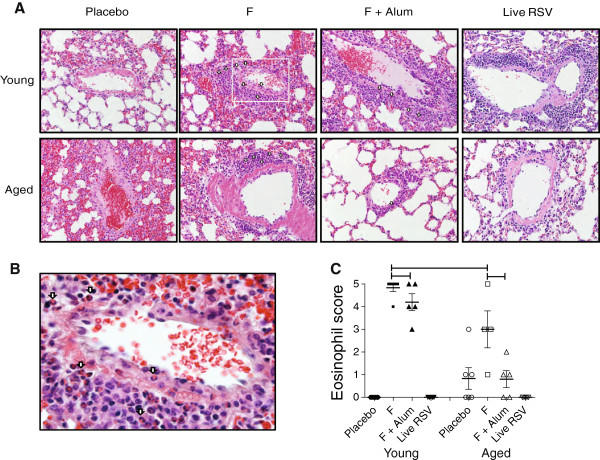
**Local accumulation of eosinophils in young and aged mice.** Vaccinated young and aged BALB/c mice were challenged with 10^6^ PFU RSV A2 at day 28. (**A**) Lungs were harvested 4 days post-challenge, stained with H&E, and eosinophil infiltration was scored. Representative sections are shown. Arrows indicate eosinophils. (**B**) The boxed inset in Figure 4A shows eosinophils (arrows) at higher magnification. (**C**) Eosinophil score ± SEM. Each symbol represents one mouse. (Brackets, *P* < 0.05, ANOVA).

We determined histopathologic changes induced by non-adjuvanted F or F + alum vaccination followed by *wt* RSV challenge in young and aged mice. While few eosinophils were observed in placebo and live RSV groups of young and aged mice following challenge, significant eosinophilic infiltrates were observed in young mice vaccinated with non-adjuvanted F or F + alum, and in aged mice vaccinated with non-adjuvanted F (Figure 4). The numbers of lung infiltrating eosinophils were significantly lower in aged mice compared with young mice (Figure 4C). In addition to the eosinophilic infiltrates, we measured other histological features including interstitial pneumonia and perivascular edema in lungs of young and aged mice. However, there were no significant differences in those parameters between the treatment groups (data not shown). Collectively, the score for infiltrating eosinophils in F + alum or non-adjuvanted F protein vaccinated aged mice was significantly lower compared to the vaccinated young mice following *wt* RSV challenge (Figure 4C). Further, the eosinophil scores were consistent with the IL-5 and eotaxin levels in the lungs of young and aged mice (Figure 3B and 3C).

## Discussion

Relatively few published studies have delineated the quality or the magnitude of the humoral and lung cytokine response to RSV in a genetically-defined aged host. Zhang *et al.* demonstrated that aged BALB/c mice have deficient RSV-specific CD8^+^ cytolytic T cell responses and IFNγ production, but RSV antigen-specific humoral responses, histopathologic changes, and viral loads were not evaluated in this study [23].

Purified RSV fusion (F), attachment (G), and matrix (M) proteins have been developed as subunit vaccines; however, RSV subunit vaccines have been plagued with poor immunogenicity in the elderly [6-11]. These studies indicated a need for investigating the ability of a highly pure RSV F preparation to induce immune responses in a genetically defined aged host. Based on these and other similar findings in the field we decided to explore if an adjuvant-driven, respiratory virus antigen-specific immune response could alleviate or correct the immune defects observed in the lungs of aged mice. In order to address the above question, we expressed a recombinant RSV F protein in mammalian cells and purified it to > 95% purity using immuno-affinity methods. Properties such as protein folding, aggregation and integrity of the antigenic sites in the purified RSV F protein were carefully characterized using a variety of biophysical and biochemical techniques that included ELISA, SDS-PAGE, Western blotting and electron microscopy. Furthermore, the alum-adjuvanted RSV F formulation was characterized using ELISA and Western blotting to ensure the preservation of the RSV F antigenic sites.

Recently published work has elucidated the possible *in vivo* mechanism by which alum mediates its adjuvant activity [24]. The authors demonstrated that in mice, alum causes cell death and the subsequent release of host cell DNA acts as a potent endogenous immunostimulatory signal mediating the induction of IgG1 and T_H_2 responses [24]. The induction of serum IgG1 antibodies by alum observed in our studies (Figure 2B) may be attributable to this damage-associated molecular pattern recognition of the host cell DNA, mediating adjuvant activity on humoral immune responses. Another study describing the potential molecular mechanism of alum activity demonstrated that alum directly interacts with dendritic cell membrane lipids, triggering signaling cascades that promote CD4^+^ T cell activation and humoral immune responses [25].

In this study, we evaluated the effect of alum adjuvant on host immune responses against RSV F protein in an RSV seronegative immunosenescent setting. Both serum neutralizing antibody titers and lung cytokine production in response to vaccination and *wt* RSV challenge were found to be diminished in aged mice compared to young mice (Figure 1 and Figure 3). However, lung eosinophilic inflammation following vaccination and challenge was also lower in aged mice compared to young mice, and F + alum vaccination did not induce additional immunopathologic lesions in aged mice relative to the placebo group (Figure 4). The relatively low eosinophilic infiltrates observed in the placebo and live RSV-infected groups (Figure 4) supported the notion that lung immunopathology is caused by the host’s inflammatory immune response to viral replication rather than being a direct response to the viral load found in the lungs following *wt* RSV challenge [26].

Our findings from the studies presented here were consistent with the lack of evidence for vaccine-mediated disease enhancement in this model reflecting the low risk of inducing a severe inflammatory response to alum immunization in aged hosts. Despite lower immune responses, F + alum vaccinated aged mice produced significant neutralizing antibodies to RSV and achieved a 2-log reduction in lung viral titers compared to the non-adjuvanted F cohort (Figure 1). Significant RSV F-specific IgG titers were measured in the lung homogenates of the F + alum and live RSV infection groups (Figure 2C); however, the individual contributions of serum antibodies present in the blood versus those secreted by lung plasma cells could not be distinguished by our methods. Alum-adjuvanted RSV F protein reduced lung viral titers by 2-log in aged mice, though the protection conferred was suboptimal compared to that seen in young mice (3-log reduction in viral titer) (Figure 1). Taken together, our data suggest that a higher F antigen dose and/or a stronger adjuvant may be required in immunosenescent populations to attain an appropriate balance of protective neutralizing antibodies and effective T_H_1-dominant cytokine responses along with minimal lung immunopathology.

Our model evaluated humoral and cytokine responses induced by *wt* RSV A2 intranasal infection and boosted by challenge. Interestingly, this group was the only one among all of the treatment groups that generated statistically significant lung T_H_1 cytokine responses (Figure 3). Additionally, RSV F-specific IgA antibodies were only detected in the lung homogenates of the live RSV-infected animals, pointing to a role for mucosal antibodies in mediating protection against natural RSV infection in both young and aged mice. The low serum neutralizing antibody titers measured in the live RSV-infected groups (Figure 1B) further supported our conclusion that protection against viral challenge was mediated by RSV F-specific IgA antibodies and/or IFN-γ responses induced in the lungs of the animals in this group. The relationship between the elicited mucosal antibody response at the local site of infection and protection against viral challenge in this group may have important implications for RSV disease in the elderly and deserves further investigation. However, the detailed evaluation of a vaccine-specific immune response in a RSV-seropositive, aged mouse model as it relates to vaccine responses induced in elderly humans was outside the scope of this study. The characterization of immune responses in a RSV-seropositive vaccinated aged mouse model is expected to provide additional mechanistic insight, facilitating the rational design of enhanced immunization strategies against RSV disease in the elderly.

## Conclusions

In this study, we found that alum-adjuvanted RSV F protein was protective in aged mice, though the protection was sub-optimal compared to that seen in young mice. Overall, day 27 serum neutralizing antibody titers and lung cytokines produced in response to vaccination and RSV challenge were diminished in aged mice compared to young mice. However, F + alum vaccination did not induce additional immunopathology in aged mice relative to vaccination with unadjuvanted F protein following RSV challenge. Despite lower immune responses, F + alum vaccinated aged mice produced significant neutralizing antibodies against RSV and achieved a 2-log reduction in lung viral titers compared to the unadjuvanted F cohort. Taken together, these data suggest that a higher F antigen dose and/or a stronger adjuvant may be required in immunosenescent populations to attain an appropriate balance of protective neutralizing antibodies, effective T_H_1-biased cytokine response and minimal lung immunopathology.

## Methods

### Mice

Pathogen-free, 8-week- and 18-month-old, female BALB/c mice were purchased from the National Institute of Aging (NIA, Bethesda, MD). All animal procedures were conducted according to the guidelines of the Institutional Animal Care and Use Committee.

### RSV antigens

The fusion subunit F protein (sequence containing amino acids 1–524) of RSV A2 was expressed in Chinese Hamster Ovary (CHO) cells (ATCC, Manassas, VA) and immuno-affinity purified with anti-RSV F monoclonal antibody (Palivizumab, MedImmune, Gaithersburg, MD) to > 95% purity. The antigenic sites of the protein were preserved as determined by an ELISA sandwich assay. Purified RSV F protein was used for both animal immunizations and coating in ELISA assays. Our data suggested that any anti-CHO host cell protein response generated in young or aged mice was not detectable in an ELISA assay using 0.5 μg/mL coating concentration of RSV F. Wild-type (*wt*) RSV A2 and Green Fluorescent Protein (GFP)-tagged RSV A2 were grown and titrated in Vero cells. Virus stocks were stabilized in sucrose-phosphate (1X SP) buffer (0.2 M sucrose, 0.0038 M KH_2_PO_4_, 0.0072 M KH_2_PO_4_), snap-frozen and stored at −70°C.

### Study design

All mice were primed at day 0 and boosted at day 14 intramuscularly (i.m) in both quadriceps. Placebo groups were given 100 μl of phosphate buffered saline (PBS), while F vaccine groups received 0.3 μg of F protein. This dose was based on our previous observation that 0.3 μg of F without adjuvant was only partially protective upon *wt* RSV A2 challenge (unpublished results). For RSV F + alum group, mice were given 0.3 μg of F protein mixed (by vortexing for 30 min at RT) with 100 μg of Alhydrogel (Aluminum Hydroxide, Brenntag Biosector, Denmark). Lastly, for the live RSV group, mice were intranasally (i.n.) infected on day 0 with 10^6^ PFU *wt* RSV strain A2. Protection was assessed by intranasal inoculation of all mice with 10^6^ PFU RSV strain A2 on day 28. Body weight was measured daily following *wt* RSV challenge until animals were euthanized.

### Serum collection

Peripheral blood was collected from the submandibular vein 27 days post-priming. Collected blood was left at room temperature (RT) for 20 min. The tubes were centrifuged at 7000 rpm for 10 min and sera stored at −80°C.

### Micro-neutralization assay

RSV-specific neutralizing antibody titers in mouse sera were measured using GFP-expressing RSV A2 in a micro-neutralization assay. Mouse sera from all treatment and placebo groups were heat-inactivated at 56°C for 45 min and serially diluted three-fold in growth medium. Equal volumes of the diluted sera were mixed with GFP-expressing RSV A2 virus to yield 500 PFU/well. Virus only and hyperimmune serum with a known neutralizing antibody titer were included on each plate as controls. The serum + virus (or virus only) mixture was incubated at 33°C, 5% CO_2_ for 1 h to allow time for the neutralizing antibodies in the serum to neutralize the virus particles. Confluent monolayers of Vero cells (ATCC, Manassas, VA) prepared in separate 96-well plates were infected with the GFP-expressing RSV A2 virus only or the serum/virus mixture from the above step. The infected Vero cell plates were incubated at 33°C, 5% CO_2_ for 22 h. The plates were washed and the fluorescent viral foci enumerated using an IsoCyteTM Reader (Blueshift Biotechnologies, Sunnyvale, CA). The 50% reduction in viral foci (antibody EC_50_ titers) was calculated using a 4-parameter curve fit algorithm.

### Serum IgG, IgG1, IgG2a, and Lung IgG ELISA

RSV F-specific IgG antibody titers in serum were measured using ELISA. Three sets of high binding 96-well plates intended to measure IgG, IgG1, and IgG2a, respectively, were coated overnight at 4°C with 0.5 μg/mL of CHO-expressed recombinant RSV F diluted in PBS. Plates were washed with PBS/0.05% Tween-20 and blocked with PBS/0.05% Tween-20 containing 0.5% bovine serum albumin (BSA, EMD Biosciences, Gibbstown, NJ) for 1 h at 37°C. The samples and hyperimmune serum reference standard (MedImmune, Mountain View, CA) were serially diluted 2-fold in PBS/0.05% Tween-20/0.5% BSA following an initial pre-dilution of 1:50. After washing, the pre-diluted samples were added to the three plates and the plates incubated at 37°C for 1 hour. Horseradish peroxidase (HRP)-conjugated, goat anti-mouse IgG, IgG1, or IgG2a antibodies (Jackson ImmunoResearch, West Grove, PA) diluted 1:20,000 in PBS/0.05% Tween-20/0.5% BSA were added, respectively, to the three plates. After washing, the plates were then developed with 3,3´,5,5´-tetramethylbenzidine (TMB, Sigma, St. Louis, MO) and stopped with 1N HCl (Sigma, St. Louis, MO). The absorbance was measured at 450 nm on a SpectraMax plate reader and analyzed using SoftMax® Pro (Molecular Devices Inc., Sunnyvale, CA). Titers were reported as Log_2_EC_50_ determined using a 4-parameter curve fit for each sample curve.

### Viral plaque assay

Mice were euthanized at day 4 post-challenge. A Beadbeater (Biospec Products, Bartlesville, OK) was used to homogenize the lungs as previously described [27]. Lung homogenates were serially diluted and used to inoculate subconfluent HEp-2 cells in 24-well plates. After 1 h adsorption at RT on a rocking platform, the cells were overlaid with MEM/10% FBS/1% penicillin G/streptomycin sulfate/amphotericin B solution /0.75% methylcellulose. After six days, the overlay medium was removed and the cells fixed with methanol. Plaques were visualized by immunodetection as described [26].

### Cytokine quantification

Lung homogenates prepared for the viral plaque assay as described above were also evaluated in a Luminex-based cytokine profiling assay. Mouse cytokine multiplex kits, custom designed to include IL-5, IL-13, IFN-γ, RANTES, MCP-1, eotaxin, KC, IP-10, and MIP-1α were purchased from Millipore (Billerica, MA). The assay was performed according to the manufacturer’s instructions. The plates were analyzed on a Bio-Rad Luminex reader (Hercules, CA), and individual cytokine levels were expressed as pg/mL. IFNγ and IL-5 were used to represent T_H_1 and T_H_2 cytokines, respectively.

### Lung histopathology

Heart-lung blocks were harvested 4 days post-infection and fixed overnight in 4% paraformaldehyde. Lungs were transferred to 70% ethanol and then embedded in paraffin blocks as described previously [27]. Tissue sections (5 μm) were stained with hematoxylin and eosin (H&E) to assess histologic changes. H&E-stained slides were digitally scanned using a Zeiss MIRAX MIDI microscope [27]. Slides were examined and scored by a pathologist who was blinded to the experimental groups. Lymphocytes, neutrophils, macrophages, and eosinophils were assessed in peribronchiolar, perivascular, interstitial, and alveolar spaces as described [27]. For the eosinophil score, groups were assessed for severity of eosinophilic infiltrate on a scale of 0 to 5 in the peribronchiolar, perivascular, interstitial, and alveolar spaces, where 0 = no eosinophils present, 1=1-10 in area, 2=11-20, 3=21-30, 4=31-40, 5= 41–50.

## Abbreviations

RSV: Respiratory Syncytial Virus; Alum: Aluminum Hydroxide; GFP: Green Fluorescent Protein; TMB: 3,3´,5,5´-Tetramethylbenzidine.

## Competing interests

The authors declare that they have no competing interests.

## Author’s contributions

A.C, S.L (Stacie Lambert), E.S, M.M and S.L (Sujin Lee) designed the described studies; A.C, K.S (Kate Stokes), K.P, H.K. and S. L (Sujin Lee) performed the described experiments; K.S (Kaori Sakamoto) performed hispathology. All authors interpreted the described data and contributed to the writing of the manuscript. All authors read and approved the final manuscript.
